# Towards transformation of psychological assessment practices in South Africa

**DOI:** 10.4102/ajopa.v6i0.164

**Published:** 2024-09-16

**Authors:** Erica Munnik

**Affiliations:** 1 Department of Psychology, Faculty of Community and Health Sciences, University of the Western Cape, Cape Town, South Africa

## Introduction

The publication in 2023 of the sixth edition edited by Foxcroft and de Kock’s *Introduction to Psychological Assessment in the South African Context* marks 5 years since the previous edition was published. This text is regarded as a valuable resource for undergraduate and postgraduate training and is prescribed or recommended by higher education institutions. In addition to its use in training, the Health Professions Council of South Africa (HPCSA) recommends its use as preparation for intern practitioners writing the National Psychology Board examination prior to registration as professional psychology practitioners in South Africa. The popularity of the previous versions demonstrated the text’s ease of use and its value for scholars and practitioners in increasing their theoretical and practical knowledge of assessment practice. This review seeks to compare the sixth edition to previous editions and to consider if the revised text is relevant for use in the academic and the world-of-work contexts.

## Target audience and contributors

The target audience is identified as ‘students and professionals in people-orientated careers’ who need to gain a basic understanding of psychological assessment and how it is applied in different contexts in South Africa. The 18 chapters are written by contributors from different sectors; these include academic institutions (e.g., departments of psychology and industrial psychology), practitioners in the field, and test developers in organisations such as JVR Solutions and Psytech. The choice of contributors from these sectors ensured the inclusion of different perspectives and theoretical frameworks, providing a broad lens to explore a multifactorial subject of this nature in the text.

## Aim of the text

The book articulated a clear focus by introducing psychological assessment as a science and focussing on its application in practice. Specific reference to some of the most prominent advances in the field is made in this edition, with a clear push towards transforming assessment towards a culturally meaningful science and modern practice. This was performed by incorporating emic and/or etic approaches to test development, aimed at better understanding human behaviour and its unpredictable nature in various applied contexts.

## Format and structure

The text is written in uncomplicated academic language, with the goal of making the subject matter understandable to students and early career practitioners. The introduction in each chapter provides the main outcomes in bullet form to give a clear sense of the purpose, goals, and outcomes. Chapters also include ethical dilemma case studies and critical thinking challenges to enhance reflexive thinking and provide the opportunity to explore more comprehensively the presented content. References to ethics principles and how these impact the practitioner’s decisions are embedded in each chapter, to foster ethical practices and assist the reader to map their progress in this regard. Recommended readings and references are provided at the end of each chapter to promote further exploration on the discussed topics. This proves useful as it gives the reader the opportunity to expand their learning through the exploration of relevant sources of information to broaden and deepen their fund of knowledge on the specific topics under discussion.

## Synopsis of content: Zones, chapters, and main outcomes

The chapters are divided into three zones. The Foundation zone, Chapters 1–7, the Assessment Practise zone, Chapters 8–9 and 15–18, and the Types of measures zone, Chapters 10–14.

The Foundation zone’s primary focus is on the theoretical foundations and more scientific aspects (the psychometrics) of assessment. Of particular interest in Chapter 1 is a discussion of the impact of coronavirus disease 2019 (COVID-19) on test development, training, and assessment practices; for example, it changed the way that assessments were taught and applied because of social distancing restrictions. These changes are expanded upon in Chapters 8, 9, 11, 14, and 18. In Chapter 2, technology-based assessments (TBA) are introduced, while Chapters 2, 6, 9, 10, 13, 14, 16, and 18 thoroughly engage with the development of TBA measures and their ethical use in South Africa.

Chapters 3–5 introduce basic measurement and the most important statistical calculations in a digestible way, with practical examples. The multifaceted steps in the development and adaptation of psychological measures, and the accompanied challenges concerning language and cultural diversity in South Africa are explored in Chapters 6–7. Emic and/or etic solutions to these challenges in measure development are further motivated in Chapter 18. The use of emic and etic approaches in measure development is an ongoing research focus in South Africa, with reference made that many such studies published in journals such as the *South African Journal of Psychology* (SAJP; https://journals.sagepub.com/home/sap), the *African Journal of Psychological Assessment* (AJOPA; https://ajopa.org/index.php/ajopa) and textbooks such as *Psychological Assessment in South Africa: Research and Applications* in Laher and Cockcroft (2019).

The ethical practice of assessment receives attention in the assessment practice zone, within the framework of statutory stipulations and practice guidelines available internationally and locally. Important issues, such as the protection of personal information, addressing linguistic factors prior to testing, and more extensive information on system-based testing are expanded upon. The use of specific tests and the advances made in research on test adaptation receive comprehensive attention in Chapters 10–14. An added advantage in this edition is that special reference is made to some of the challenges that developers experience in adapting tests for use in a multilingual and culturally diverse country, with a focus on recommendations on how to overcome these challenges in Chapters 2, 7, 10, 11, 17, and 18.

Interestingly, the need to enhance capacity-building in research on test development and adaptation becomes a recurring theme, with specific examples including the need to develop and/or adapt cognitive measures for children (Chapter 10) and personality measures for children and adolescents (Chapter 12). The use of artificial intelligence (AI) and simulations, and an extensive discussion on the advances made in the creation of TBA (Chapter 14) provided an interesting read, showcasing some of the progress made to incorporate technology in assessments, especially in industry. A caution was raised in that the role of the clinician should not be marginalised and that sufficient evidence regarding reliability and validity is needed before technology-based methods are adopted.

A brief overview of the application of assessment measures to industry and education is provided in Chapter 15, while Chapter 16 offers an overview of the importance of accurate interpretation and reporting of assessment results, essential aspects of assessment to be maintained by the practitioner. Chapter 17 focuses on factors that might impact assessment practices; these include the biological or physical aspects of the individual, the intrapsychic and interpersonal or social context, as well as the impact of language and culture on assessment practices. Environmental factors such as home environment, poverty, socio-economic status (SES), and urbanisation are also introduced as possible barriers to assessment practices. The last chapter, Chapter 18, provides an integration of the most important ‘take-home’ messages and discusses the way forward, with emphasis on some of the lessons learned in the journey towards culturally meaningful psychological assessment practices in South Africa.

## Relevance and utility of the book

In its sixth edition, this text remains a valuable and relevant guide for under- and postgraduate students and early career practitioners as an introduction to the history of assessment, as well as the theoretical foundations needed to operationalise contextually sensitive assessment in practice; it also enables students to gain an understanding of the advances made in South Africa in the field of assessment in the past few years. Many students in South Africa still enter their postgraduate programme with a lack of knowledge in psychometric assessment, as undergraduate curricula do not include exposure to psychological assessment modules. Postgraduate students are therefore afforded the opportunity to engage in this text either by means of self-study or the inclusion of the text as a prescribed source in Honours and Masters courses to increase their fund of knowledge in the area of assessment. In addition, possible shifts in the scope of practice of psychologists as considered by the HPCSA makes this text a seminal one to ensure that practitioners across registration categories have the opportunity to familiarise themselves with the basic tenets of psychological assessment.

Unique features of this text include the discussion of the many challenges that we still face in the area of assessment and the advances that have been made to date, such as a continuous focus on the design and adaptation of contextually and culturally appropriate measures. However, much still needs to be done to attend to remaining challenges in assessment practice; in particular, academics, researchers, practitioners, and industry need to combine efforts to advance the psychological assessment field. The text also raises how challenging but essential the development and adaptation of measures are to cater for the diverse needs, cultures, and multilinguistic nature of the South African context. Thus, sustained research in the field of assessment is essential to ensure that assessment measures are meaningful and relevant for our diverse population. In the words of Cheryl Foxcroft: ‘This is not the end of a journey but the beginning of a transformative one’.
